# The Effect of La^3+^ on the Methylene Blue Dye Removal Capacity of the La/ZnTiO_3_ Photocatalyst, a DFT Study

**DOI:** 10.3390/nano12183137

**Published:** 2022-09-10

**Authors:** Ximena Jaramillo-Fierro, Guisella Cuenca, John Ramón

**Affiliations:** 1 Departamento de Química, Facultad de Ciencias Exactas y Naturales, Universidad Técnica Particular de Loja, San Cayetano Alto, Loja 1101608, Ecuador; 2 Ingeniería Química, Facultad de Ciencias Exactas y Naturales, Universidad Técnica Particular de Loja, San Cayetano Alto, Loja 1101608, Ecuador

**Keywords:** DFT calculations, photocatalytic materials, lanthanum doping, adsorption mechanism, pollutant degradation

## Abstract

Theoretically, lanthanum can bond with surface oxygens of ZnTiO_3_ to form La-O-Ti bonds, resulting in the change of both the band structure and the electron state of the surface. To verify this statement, DFT calculations were performed using a model with a dispersed lanthanum atom on the surface (101) of ZnTiO_3_. The negative heat segmentation values obtained suggest that the incorporation of La on the surface of ZnTiO_3_ is thermodynamically stable. The bandgap energy value of La/ZnTiO_3_ (2.92 eV) was lower than that of ZnTiO_3_ (3.16 eV). TDOS showed that the conduction band (CB) and the valence band (VB) energy levels of La/ZnTiO_3_ are denser than those of ZnTiO_3_ due to the participation of hybrid levels composed mainly of O2*p* and La5*d* orbitals. From the PDOSs, Bader’s charge analysis, and ELF function, it was established that the La-O bond is polar covalent. MB adsorption on La/ZnTiO_3_ (−200 kJ/mol) was more favorable than on ZnTiO_3_ (−85 kJ/mol). From the evidence of this study, it is proposed that the MB molecule first is adsorbed on the surface of La/ZnTiO_3_, and then the electrons in the VB of La/ZnTiO_3_ are photoexcited to hybrid levels, and finally, the MB molecule oxidizes into smaller molecules.

## 1. Introduction

Among the various industries, the textile industry is characterized by the generation of significant amounts of wastewater, in which dyes are some of the main components [1,2]. The dye methylene blue (MB) is widely used in textile products, making it one of the most common water contaminants [3]. Although many strategies, such as filtration and adsorption, have been proposed for the decontamination of aqueous systems, an economical and environmentally friendly procedure to purify large amounts of wastewater is still required [4,5]. Today, advanced oxidation processes (AOPs), including the Fenton method, Fenton-like method, and photoelectric catalytic processes, have gradually emerged due to their efficiency in removing organic contaminants [6]. Photocatalysis is also an AOP; therefore, it represents an interesting alternative for the degradation of organic pollutants, taking advantage of highly reactive substances as well as a light source [7,8]. In fact, the use of photocatalytic materials that have the capacity to produce reactive oxygen species (ROS) in the presence of sunlight could be one of the most accessible alternatives, since the sun is a suitable source of energy to promote chemical reactions without cost [9].

Technological development has allowed the design of various semiconductors with potential application in photocatalytic processes, including metallic compounds, such as oxides, chalcogenides, nitrides, and oxyhalides, among others. Photocatalytic semiconductors absorb photons to generate electrons (*e*^−^) and holes (*h*^+^), which are then required to promote oxidation–reduction chemical reactions. An efficient photocatalyst should generally have the following attributes: appropriate bandgap energy, large light absorption range, and high charge migration capacity, as well as good stability, strong catalytic activity, and, from an economic view, high sustainability and low cost [10].

Zinc and titanium oxides are of great interest due to their ready availability, relatively low cost, minimal toxicity, good stability, and excellent ability to produce strongly oxidizing radical species [11,12,13,14]. Simultaneous preparation of the ZnO/TiO_2_ compound generally allows for the formation of three other well-known compounds, including perovskite ZnTiO_3_ (hexagonal, cubic), spinel Zn_2_TiO_4_ (cubic, tetragonal), and defect spinel Zn_2_Ti_3_O_8_ (cubic) [15,16,17]. Among them, the ZnTiO_3_ perovskite type has received much attention in photocatalytic reactions due to its relatively low cost, wide bandgap, compositional flexibility, unique chemical and physical properties such as high structural and thermal stability, and electron transport properties [18,19,20,21].

ZnTiO_3_ is a novel polar oxide containing Zn^2+^ (3*d*^10^) and Ti^4+^ (3*d*^0^), which have colombic repulsion with each other [22,23]; therefore, ZnTiO_3_ is a potential candidate as a ferroelectric, nonlinear optical, and piezoelectric material [24,25]. Furthermore, this semiconductor shows promise for the fabrication of dye-sensitized solar cells and for the treatment of harmful organic compounds by photocatalytic remediation [26,27,28,29,30].

Doping methodology can be a powerful approach to change the structural and catalytic properties of semiconductor oxides [31,32]. The dopant metal is often adapted to respond to visible light, so hybrid energy levels can form between the valence band (VB) and the conduction band of such oxides [33]. Therefore, proper doping can prevent photoinduced electron–hole (*e^−^/h^+^)* pair recombination and improve the photoactivity of semiconductor oxides, including perovskite ZnTiO_3_ [34,35].

The photoactivity of semiconductors can be improved through the doping technique using different group of elements, such as metals (Au, Cu, Ag, Co, etc.) [36], non-metals (Mg, N, C, S, etc.) [37], and rare earth metals (Ce, Eu, La, etc.) [38]. Rare earth metals such as lanthanide ions are potential candidates for doping since they have 4*f* levels in their electronic configuration, which could act as effective reservoirs of electrons to trap them in the conduction band (CB) of the photocatalyst [39,40]. In addition, doping with lanthanide elements can improve light sensitivity and facilitate the transport and diffusion of reactive species and products by increasing the adsorption capacity of the adsorbate [40].

Several experimental and theoretical investigations have shown that the optical and electronic properties of materials can be improved by incorporating rare earth (RE) dopant ions [41], especially the lanthanum ion, due to its electron-trapping effect provided by the adaptable chemical valence sites (La^2+^ and La^3+^). Lanthanum (La) is one of the rare earth metal elements widely investigated for various purposes, due to its attractive properties and natural abundance (100%) [42]. Lanthanum is an effective dopant for modulating the band structure and enhancing the photocatalytic properties of various semiconductor oxides, including ZnTiO_3_ [43,44,45].

Various authors indicate that the La-doped semisconductor has completely different physical–chemical properties compared to the undoped semiconductor [46]. The presence of La in the ZnTiO_3_ lattice generates electron capture and therefore prevents their recombination with holes to limit the formation of electron–hole pairs (*e*^−^/*h*^+^), which leads to an improvement in the photochemical efficiency of this semiconductor [47]. Moreover, the presence of La in the ZnTiO_3_ lattice reduces its bandgap and changes its optical behavior to the visible region, which improves the photoactivity of the semiconductor under sunlight [48].

However, a higher adsorption capacity due to a higher concentration of lanthanum is not a condition for a higher photocatalytic activity, which may be limited by a lower separation rate of the photoinduced (*e*^−^/*h*^+^) pairs. In fact, an excessively high concentration of lanthanum could increase the amount of oxygen vacancies on the surface, which can become photoinduced electron–hole (*e*^−^/*h*^+^) pair recombination centers [49]. Since electron–hole separation has a more important protagonist than adsorption capacity in photocatalytic processes, several studies have reported that a concentration of 1–2% (*w*/*w*) of lanthanum in the lattice of a photocatalyst facilitates charge transfer between the VB or CB of the photocatalyst and the *d*/*f* levels of the La^3+^ ions that were incorporated into the respective lattice. An increase in lanthanum concentration would result in a high accumulation of nanoparticles, which can protect the photocatalyst surface from light absorption and lead to lower photocatalytic activity.

The literature suggests that the La^3+^ ion cannot replace the Ti^4+^ or Zn^2+^ ions of the lattice in the ZnTiO_3_ body, since the ionic radius of the La^3+^ ion (1.15 Å) is much larger than that of Ti^4+^ (0.68 Å) or the Zn^2+^ ion (0.74 Å) [44], and the charge of La^3+^ does not coincide with the charge of Ti^4+^ or Zn^2+^ [40,46]. However, the La^3+^ ion can be well dispersed on the surface of ZnTiO_3_ semiconductor particles, forming Ti-O-La bonds with ionic-covalent characteristics [50].

The dispersion of La^3+^ on the surface of ZnTiO_3_ can cause lattice distortion in the surface layer and the generation of defects that block the growth of the catalyst crystallites and increase the number of oxygen vacancies in the catalyst [47,49,51]. On the other hand, the great diameter of La^3+^ is useful for improving the specific surface area (SSA) of ZnTiO_3_, and since La^3+^ shows a strong electron-withdrawing effect, the presence of this ion could also contribute to the formation of Lewis acid sites [52], which would improve the adsorption of organic contaminants on the photocatalyst surface [40].

The experimental results obtained previously showed that the incorporation of La in the lattice of the coupled semiconductor ZnTiO_3_/TiO_2_ (ZTO) induced the narrowing of its bandgap and also promoted the photocatalytic activity of the coupled semiconductor, probably due to the ability of La^3+^ ions to catch electrons in the CB of the ZTO and consequently increase the lifetime of photogenerated charge carriers. In addition, the La-ZTO nanocomposite sample was found to have a higher SSA and smaller average crystallite size compared to the corresponding pure ZTO. The average crystallite size of the nanocomposite reduced with the lanthanum incorporation, probably because when La^3+^ ions occupy the regular sites of the ZTO lattice, they form structural defects that block crystallite growth. On the other hand, UV–Vis diffuse reflection spectroscopy (UV–Vis XRD) studies showed that, compared to the pure ZTO, the absorption edge of the La-ZTO exhibits a slight redshift, which could be associated to the charge transition between the 4*f* electrons of lanthanum and the CB of the coupled semiconductor ZTO [53].

Despite these results, to date there is no systematic study at the molecular level of the effect of the La^3+^ ion on the electronic properties of the semiconductor La/ZnTiO_3_ nor of the synergistic effect of this ion on the adsorption capacity and photoactivity of the semiconductor for its application in the removal of methylene blue (MB) dye under solar light. Density Functional Theory (DFT) is a widely used computational method for calculating the electronic structure of an isolated molecule at the quantum level. DFT allows obtaining results with the desired chemical precision as long as a sufficiently large set of bases is used, the electronic correlation is adequately described, and relativistic effects are correctly included in the calculation [54]. Therefore, the objective of this study is to use computational calculations of the Density Functional Theory (DFT) to explain the changes produced in the electronic structure of ZnTiO_3_ due to doping with lanthanum and thus propose a mechanism for adsorption and photocatalytic degradation of the MB molecule on the surface (101) of both ZnTiO_3_ and La/ZnTiO_3_.

The results presented in this computational study are an original contribution to current knowledge, since they explain for the first time at the molecular level the effect of the lanthanum ion on the electronic properties of La/ZnTiO_3_, as well as the improvement of adsorption and photocatalytic properties of the La-doped catalyst for its potential use in removing MB dye under solar light.

## 2. Materials and Methods

Density Functional Theory (DFT) study was developed using the Vienna Ab Initio Simulation Package (VASP) version 6.0 (VASP Software GmbH, Vienna, Austria) [55,56]. For modeling and visualization of the corresponding molecules and structures, the molecular modeling program BioVia Materials Studio, version 5.5 (BioVia, San Diego, CA, USA) was utilized. The ionic potential of inner nuclei and electrons was described by pseudopotentials following projector augmented wave (PAW) approach [57]. All calculations were executed using the Perdew–Burke–Ernzerhof (PBE) generalized gradient approximation (GGA) functional to describe the electronic exchange-correlation interactions [58]. The plane wave cutoff energy was brought to a value of 500 eV. The Kohn–Sham equations [59] were solved in a self-consistent way allowing the variation in energy between cycles to be less than 10^−5^ eV.

The MB molecule adsorption on the surface of La/ZnTiO_3_ was modeled using the following previously optimized parameters: hexagonal ZnTiO_3_ with a cell = 5.15 Å × 5.15 Å × 13.94 Å <90° × 90° × 120°> [60]. Composite properties were estimated by sampling the first Brillouin zone by means of Monkhorst–Pack [61] *k*-point meshes of 3 × 2 × 1. All calculations were non-spin polarized. To improve the convergence of the total energy, the Gaussian smearing method was used with σ = 0.10 eV. All atomic positions relaxed completely until the respective forces were less than 0.001 eV/Å.

The valence electron configuration for the lanthanum (La) atom was 5*s*^2^5*p*^6^5*d*^1^6*s*^2^. The functional GGA+U was also used in the calculation of the structures, since the DFT+U (GGA+U) approach has proven to be useful in correcting some of the deficiencies and also shows promise in studies of *d*-electron systems [62]. Hubbard U values were established at 2.5 eV and 6.0 eV for Ti and La atoms, respectively [26,44].

The bulk of ZnTiO_3_ was cleaved at the stable surface (101) [63,64,65] to study MB adsorption. The La/ZnTiO_3_ (101) slab model consisted of a p(2 × 3) supercell, with 36 Zn atoms, 36 Ti atoms, 108 O atoms, and 1 La atom. The values of the surface energies (*γ_s_*) of the La/ZnTiO_3_ structure with a vacuum distance of 20 Å were estimated using the following equation [50,66]:

(1) $$\gamma_{s}=\frac{\left(E_{slab}-n\times E_{bulk}\right)}{2A}$$

where $E_{slab}$ is the total energy of the slab material (eV), $E_{bulk}$ is the total energy of the bulk material (eV), *n* is the number of atoms involved in the slab, and *A* is the surface area (Å^2^).

On the other hand, the adsorption energy ($\Delta E_{ads}$) of the MB molecule on the surface (101) of both ZnTiO_3_ and La/ZnTiO_3_ oxides was estimated using the following equation [44,67]:

(2) $$\Delta E_{ads}=E_{sorb/surf}-E_{surf}-E_{sorb}$$

where $E_{sorb/surf}$ is the energy of the supersystem produced by the adsorbed molecule on the surface (eV), $E_{surf}$ is the energy of the surface (eV), and $E_{sorb}$ is the energy of the isolated molecule in vacuum (eV).

In this study, the adsorption of the La atom on the surface (101) of ZnTiO_3_ was investigated as follows. First, the clean surface of the semiconductor was subjected to the relaxation process. During this process, one layer of surface atoms was allowed to relax freely while the other atoms were immobilized. Then, a La atom was adsorbed on the surface (101) of ZnTiO_3_, which coordinated with the O atoms on the surface. The surface with a coordinated La atom also underwent relaxation. On the other hand, the adsorption of the methylene blue (MB) molecule on the surface (101) of La/ZnTiO_3_ was investigated by placing the MB molecule with different orientations. First, the P_0_ orientation was tested by placing the MB molecule in a vertical position, with the terminal methyl groups oriented to the coordinated lanthanum atom on the semiconductor surface. For the P_1_ and P_3_ orientations, the MB molecule was placed completely parallel, bringing the S and N heteroatom closer to the coordinated lanthanum atom on the semiconductor surface. In contrast, for the P_2_ and P_4_ orientations, the MB molecule was placed partially parallel, with the S or N heteroatom close to the coordinated La atom on the semiconductor surface, respectively.

Furthermore, to investigate the influence of La on molecular adsorption stability, values of heat of segregation ($\Delta G_{seg}$) relaxation were calculated. The heat segregation ($\Delta G_{seg}$) can be obtained by means of the following equation [44]:

(3) $$\Delta G_{seg}=\frac{1}{n}(E_{MB/oxide:nLa}-E_{MB/oxide}-n\mu_{Het}+n\mu_{La})$$

where $E_{MB/oxide:nLa}$ and $E_{MB/oxide}$ are the total energies of the surfaces with and without La, and n is the number of the La atoms on the surface. *μ* is the chemical potential of the heteroatom (N or S) of the MB ring. In general, a more negative value of $\Delta G_{seg}$ is evidence that the surface is thermodynamically more stable.

## 3. Results

### 3.1. Optimization of La/ZnTiO_3_

**Figure 1:**
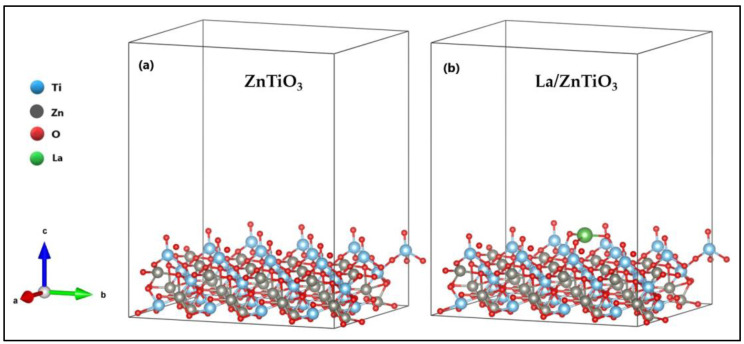
Optimized surface (101) of (**a**) ZnTiO_3_ and (**b**) La/ZnTiO_3_.

The structure of ZnTiO_3_ with a subjacent hexagonal pattern with a rhombohedral-centered hexagonal Bravais lattice and R-3(148) space group [24,26] was optimized in a previous study [68]. The surface (101) of both ZnTiO_3_ and La/ZnTiO_3_ after relaxation is shown in Figure 1a and 1b, respectively.

As can be seen, the La atom formed bonds with three O atoms on the ZnTiO_3_ surface. The three surface O atoms moved toward the La atom, away from the adjacent Ti atoms just below, consequently increasing their initial bond lengths by about 0.09 Å to generate the new La-O bonds. The O-Ti bond lengths on the ZnTiO_3_ and La/ZnTiO_3_ surfaces are comparatively shown in Table S1.

La-O bond lengths ranged from 2.32 Å to 2.38 Å with an average of 2.35 Å and La-O-Ti angles ranged from 116.25° to 133.26° with an average of 122.18°, so there are no obvious symmetry elements inside the La polyhedron. The location of the shortest and longest La-O bonds in the structure confirms that it is not easy to adjust a sphere to the oxygen sites. Similar geometric distortions have been reported by other authors [69]. Significant bond lengths and angles on the surface of La/ZnTiO_3_ are shown in Table 1.

On the other hand, the surface energy of the surface (101) of the La/ZnTiO_3_ structure was obtained by Equation (1). The surface energy value (*γ_s_*) of the surface (101) of La/ZnTiO_3_ with a vacuum distance of 20 Å was estimated to be 0.069 eV/Å^2^ (1.10 J/m^2^). This value is similar to the previously reported surface energy value for the surface (101) of ZnTiO_3_, which was 0.076 eV/Å^2^ (1.21 J/m^2^).

The adsorption energy (Δ*E_ads_*) of the La atom on the surface (101) of ZnTiO_3_ oxide after relaxation was calculated using Equation (2), where $E_{sorb/surf}$ is the energy of the supersystem generated by the adsorbed La atom on the ZnTiO_3_ surface (eV), *E_surf_* is the energy of the clean oxide (eV), and *E_sorb_* is the energy of the isolated La atom in a vacuum (eV). The adsorption energy value of the La atom on the surface (101) of ZnTiO_3_ was calculated to be −852.46 kJ/mol. The adsorption of the La atom involved the formation of new chemical bonds, which suggests that a chemisorption process occurred [70].

### 3.2. Electronic Structure of La/ZnTiO_3_

**Figure 2:**
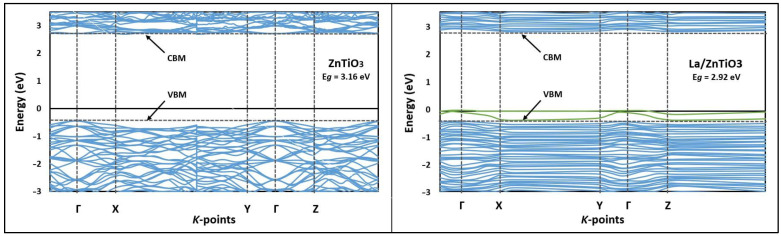
Band structure of ZnTiO_3_ and La/ZnTiO_3_ along the high symmetry directions in the Brillouin zone.

The set of lines and points of high symmetry in the first Brillouin zone [71] and the results obtained from the estimation of the electronic band structure of La/ZnTiO_3_ are shown in Figure 2. In this figure, it is also shown that La/ZnTiO_3_ has two impurity energy levels (green lines) located just above the valence band maximum (VBM) of pure ZnTiO_3_. Furthermore, Figure 2 shows that the indirect bandgap energy value of the ZnTiO_3_ and La/ZnTiO_3_ structures calculated by the GGA+U method were 3.16 eV and 2.92 eV, respectively.

**Figure 3:**
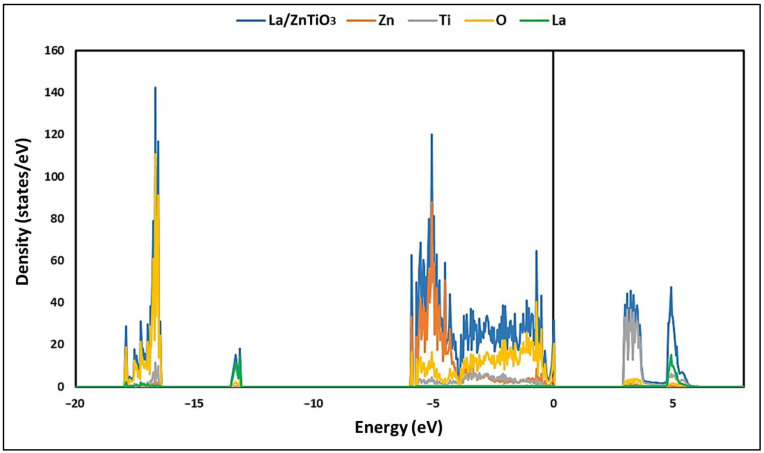
Total density of states (TDOS) of La/ZnTiO_3_.

On the other hand, the density of states (DOSs) was determined to provide more information about the bonds present in the La/ZnTiO_3_ structure [22]. Figure 3 shows the total density of states (TDOSs) of La/ZnTiO_3_. The energy levels constituting the conduction band (CB) and the valence band (VB) are slightly denser than those previously reported for pure ZnTiO_3_, probably due to the participation of hybrid levels, composed mainly of O2*p* and La5*d* orbitals [44].

The TDOS of La/ZnTiO_3_ has two principal zones: a lower valence band (VB) zone, from about −18 to 0 eV, and an upper conduction band (CB) zone from about 2 to 6 eV. The VB is affected by the contribution of Zn and O, while the CB is affected by the contribution of Ti and La. In the La/ZnTiO_3_ structure, the valence band maximum (VBM) is bordered by the O atom, while the Ti atom establishes the conduction band maximum (CBM).

**Figure 4:**
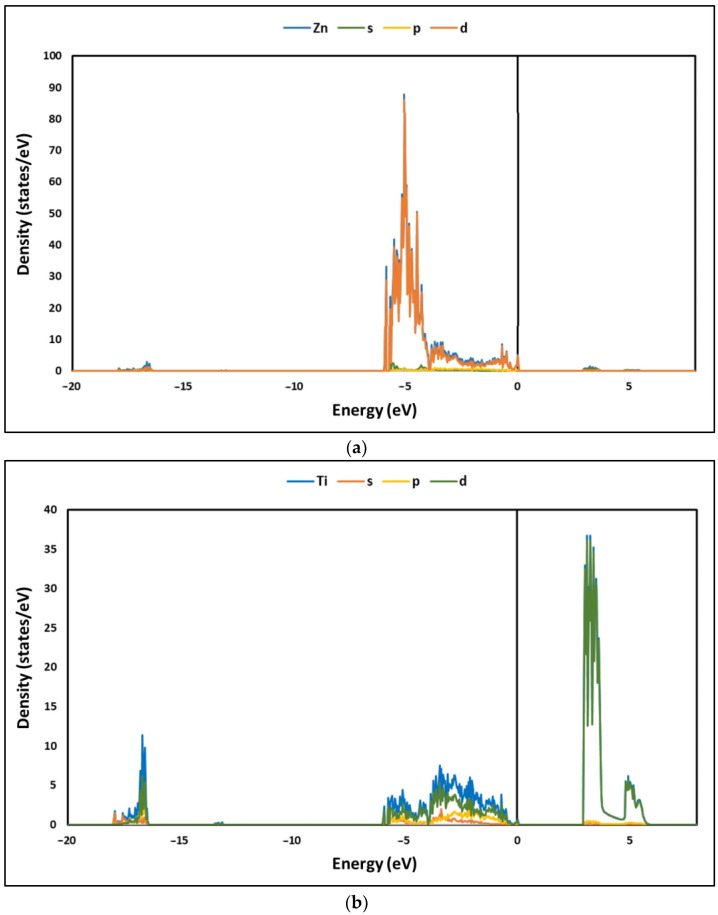

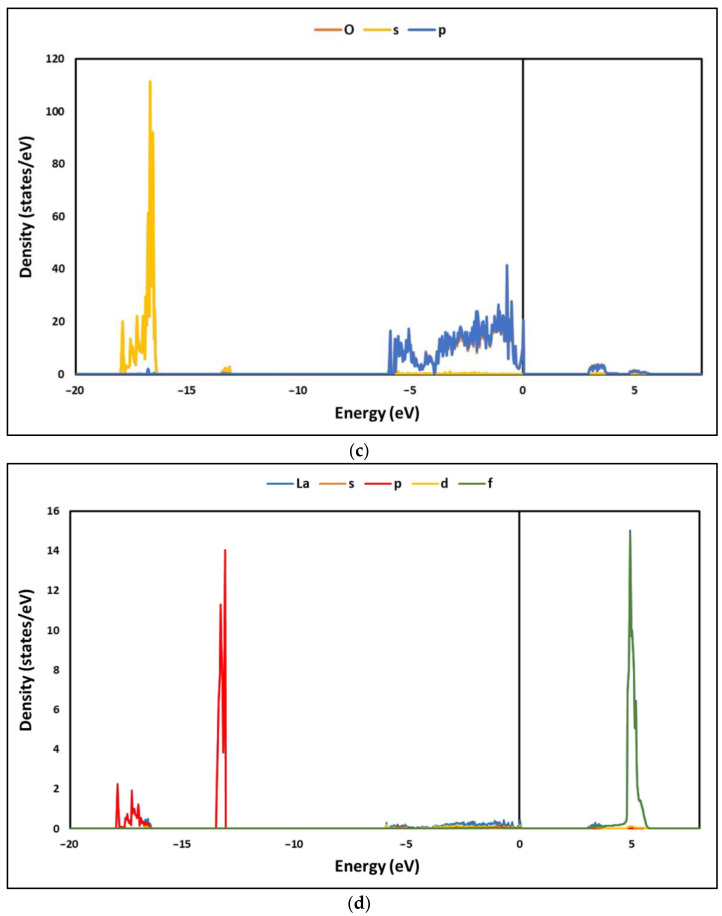
Partial density of states (PDOS) of (**a**) Zn, (**b**) Ti, (**c**) O, and (**d**) La.

Furthermore, Figure 4a–d show the partial density of states (PDOSs) of La/ZnTiO_3_. From these figures, it is evident that in the energy range from −18 to −16 eV, the TDOS arises principally from the O2*s* orbital, with a small contribution from the Zn3*d* orbital and the Ti3*p*, Ti3*d*, and Ti4*s* orbitals. In the energy region from −6 to 0 eV, TDOS arises principally from the Zn3*d*, Ti3*d*, and O2*p* orbitals, and there are obvious hybridizations between the Zn3*d*–O2*p* and Ti3*d*–O2*p* orbitals. Above the Fermi level, in the energy region from 2 to 6 eV, the total DOS arises principally from the Ti3*d*, Zn3*d*, and O2*p* orbitals. These results agree with those informed in the literature [24]. Figure 4c,d show the PDOS of O and La atoms on the surface of La/ZnTiO_3_. Two types of hybridizations are evidenced from the PDOS results. First, the overlap of the La5*d* and O2*p* states is observed in the energy region from −6 to 0.1 eV. The wide range of states indicates that the La5*d* and O2*p* orbitals become delocalized, which could indicate ionic bonding. Second, there is also an overlap of La5*p* and O2*s* states in the range −18 to −16 eV and around −13 eV. These latter peaks are relatively narrow, suggesting that the La5*p* and O2*s* orbitals are both localized. The presence of localized electrons is a common characteristic of covalent bonding. The results show that the La-O bond is probably a combination of the ionic and covalent bonds (polar covalent bond). These results agree with those informed in the literature [50].

In order to verify the chemical features of the La-O bond described above, population analysis of La/ZnTiO_3_ was also performed using the Bader’s method [72]. This analysis is useful since the ionicity of a bond can be described in terms of charge transfer between the atoms that form the chemical bond [73,74]. In the La/ZnTiO_3_ structure, the net charge of Ti (+2.5*e*) was 1.5*e* less than its formal +4*e* charge, while the Zn atom had a positive charge of +1.3*e* and the O atom had a negative charge of −1.3*e*, being in both cases 0.7*e* less than their respective formal charges +2*e* and −2*e*. Finally, the net charge of La (+2.2*e*) was 0.8*e* lower than its formal charge of +3*e*. These results agree with those described in other studies [75]. The coordinates and the Bader’s charge analysis of the optimized ZnTiO_3_ and La/ZnTiO_3_ surfaces are provided in Table S2. According to the literature, a nonzero Bader charge transfer indicates a bond ionicity of zero; likewise, a very small charge transfer of about 0.05*e* between bonding atoms indicates a weak bond ionicity. Finally, a large charge transfer (e.g., 1.5*e*) indicates significant bond ionicity [24,73]. In this study, an increase in the magnitude of the net charge of the three surface oxygen atoms that gave rise to the new La-O bonds was observed, as well as a decrease in the magnitude of the net charge of the respective lanthanum atom. Therefore, in agreement with the literature, the results of the Bader’s analysis suggest the slight ionization of the La-O bond due to a possible charge transfer between the atoms that form the chemical bond [73,74].

**Figure 5:**
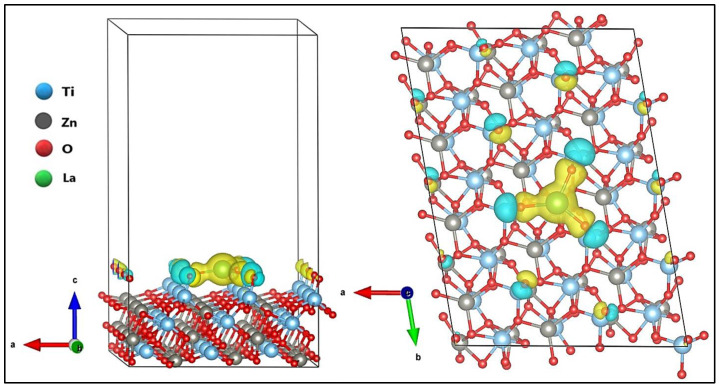
Charge density difference after La doping on ZnTiO_3_.

Charge difference analysis was used to measure the charge redistribution on the ZnTiO_3_ surface due to lanthanum doping [73]. The results of the charge redistribution in the La/ZnTiO_3_ structure are shown in Figure 5. In this figure, the evident interaction between the three surface oxygen atoms of ZnTiO_3_ and the lanthanum atom suggests that the adhesion of La-O would be mainly influenced by the charge transfer between the La and the surface oxygens. The cyan and yellow surfaces correspond to the regions of charge gain and loss, respectively, which supports Bader’s analysis above.

**Figure 6:**
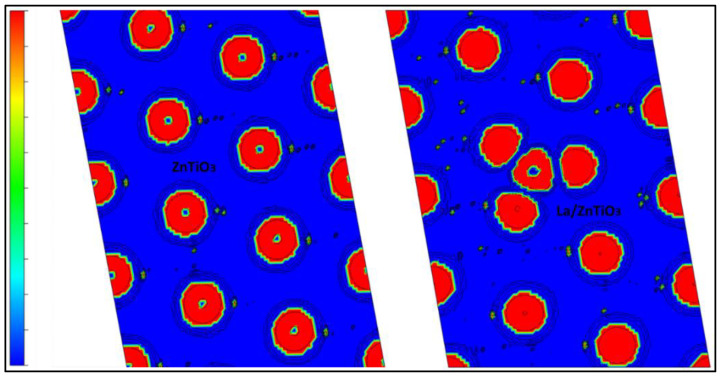
Representation of ELF with contour lines of ZnTiO_3_ and La/ZnTiO_3_ surfaces.

Moreover, the electron localization function (ELF) was utilized to better understand the La-O chemical bond [76]. In ELF analysis, when the region of maximum density (RMD) is more symmetrically distributed around the nucleus, a more ionic or van der Waals interaction occurs. If the covalent character of a bond enhances, RMD migration between centers becomes more obvious until a completely symmetric geometry is achieved in the ideal covalent case. In Figure 6, the ELF section for the surface (101) of La/ZnTiO_3_ shows the interaction of the La atom with three O atoms on the surface. The figure shows that the RMDs are in the line that joins the nuclei and can be separated from the nuclei themselves by a path; in addition, the RMDs do not circumscribe the nucleus. Therefore, a polar covalence would be generated in the La-O bonds [77].

### 3.3. MB Adsorption on Surface (101) of ZnTiO_3_ and La/ZnTiO_3_

**Figure 7:**
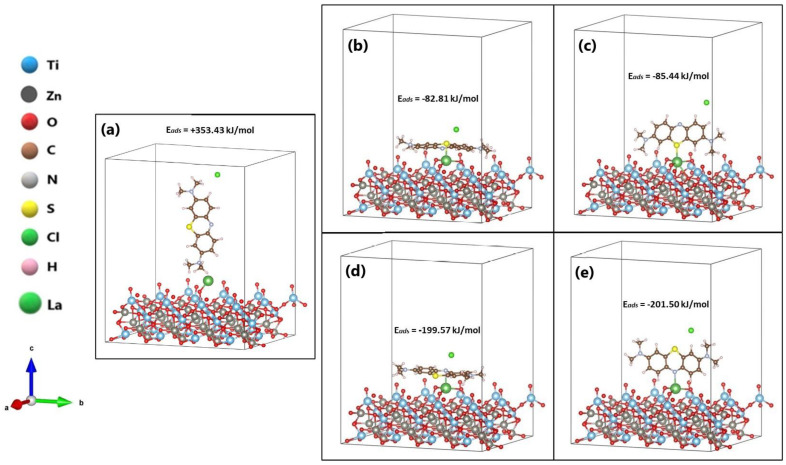
Methylene blue (MB) molecule in (**a**) P_0_, (**b**) P_1_, (**c**) P_2_, (**d**) P_3_, and (**e**) P_4_ orientations on the La/ZnTiO_3_ surface.

The tested orientations of the methylene blue (MB) molecule on the surface (101) of La/ZnTiO_3_ are shown in Figure 7. In this figure it is evident that the MB molecule is adsorbed when placed in the P_1_, P_2_, P_3_, and P_4_ orientations. However, no interaction was observed in the P_o_ orientation where the MB molecule was placed in a vertical position.

To investigate the influence of La on molecular adsorption stability, adsorption values ($\Delta E_{ads}$) after relaxation were calculated. The $\Delta E_{ads}$ of the MB molecule on the surface (101) of the La/ZnTiO_3_ oxide were calculated using Equation (2), where $E_{sorb/surf}$ is the energy of the supersystem generated by the MB molecule on the oxide surface (eV), $E_{surf}$ is the energy of the clean oxide (eV), and $E_{sorb}$ is the energy of the isolated MB molecule in a vacuum (eV). Likewise, heat segregation ($\Delta G_{seg}$) was obtained by means of Equation (3). Table 2 lists the adsorption energy ($\Delta E_{ads}$) and heat segregation ($\Delta G_{seg}$) of the different interfaces after relaxation.

**Figure 8:**
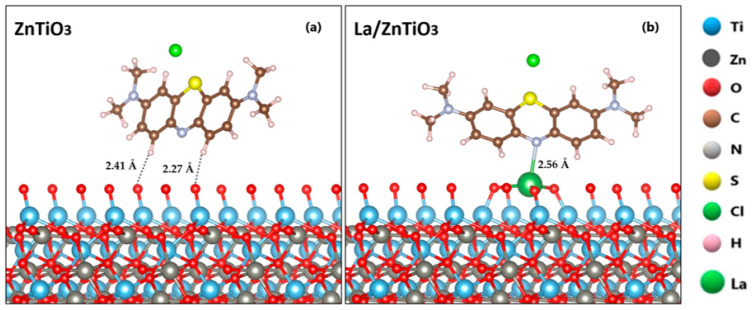
Methylene blue (MB) molecule adsorbed on the (**a**) ZnTiO_3_ and (**b**) La/ZnTiO_3_ surfaces.

Since the molecular adsorption process of MB on the surface of La/ZnTiO_3_ with the molecule located in the P_4_ orientation was energetically more favored than in the other orientations, we studied the molecular adsorption process of the MB molecule on the surface of ZnTiO_3_ only with the P_4_ orientation. As shown in Figure 8, the MB molecule is more strongly adsorbed on the La/ZnTiO_3_ surface than on the ZnTiO_3_ surface. The average distances from the H atoms of the MB molecule (H_MB_) to the surface plane of ZnTiO_3_ are H_MB_-O_(oxide)_ = 2.27 Å and H_MB_-O_(oxide)_ = 2.41 Å. The average distance from the nitrogen atom of the MB molecule (N_MB_) to the lanthanum atom on the surface plane of La/ZnTiO_3_ is N_MB_-La_(oxide)_ = 2.56 Å.

The adsorption energy value of the MB molecule on the clean surface was −64.06 kJ/mol. With the addition of La on the semiconductor surface, the adsorption energy was more stable for the adsorption of the MB molecule through the N-heteroatom of its aromatic ring (around −200 kJ/mol) compared to the adsorption of the MB molecule through the S heteroatom of its aromatic ring (about −85 kJ/mol). At the same time, the heat segregation values were around −58.19 eV and −57.00 eV for MB adsorption on the La/ZnTiO_3_ surface through the N and S heteroatoms of the MB ring, respectively. Negative values suggest that the incorporation of La on the semiconductor surface is thermodynamically stable. Therefore, it can be assumed that the presence of La increases the surface binding strength with more stable adsorption energy. Furthermore, the results imply that the P_3_ and P_4_ orientations were energetically more stable compared to the P_1_ and P_2_ orientations.

Bader’s charge analysis was also used to semiquantitatively assess charge transfer. The results of this analysis for the certain atoms at the surface of the semiconductors are listed in Table 3.

From the Bader’s charge analysis, it is evident that there was charge transfer between the MB molecule and neighboring atoms on the ZnTiO_3_ and La/ZnTiO_3_ surfaces. Furthermore, it was shown that the direction of charge transfer for the ZnTiO_3_ surface is from the MB molecule towards the catalyst, while for the La/ZnTiO_3_ surface the direction is the opposite. Likewise, Table 3 shows that there are areas of charge depletion around the H and La atoms and areas of charge accumulation around the O and N atoms, which suggests that the molecular adsorption of MB on the surfaces of ZnTiO_3_ and La/ZnTiO_3_ occurs through the formation of H-O hydrogen bonds and N-La ionic bonds, respectively.

## 4. Discussion

### 4.1. Optimization of La/ZnTiO_3_

Theoretically, lanthanum (La), due to its ionic radius, is difficult to intercalate in the ZnTiO_3_ lattice, but it can bond with the oxygen of the surface lattice to generate the La-O-Ti bond. In a previous experimental study, we found that La dispersion on the ZnTiO_3_ surface contributed to the decrease of X-ray diffraction (XRD) peak intensities [78]. This was probably due to the generation of La-O-Ti bonds on the surface of ZnTiO_3_ crystallites, which inhibited their growth by limiting direct contact with adjacent crystallites [79].

In this theoretical study, we use a model with a La atom (~2% *w*/*w*) intercalated only in the upper layer of ZnTiO_3_ to clarify the dispersion mechanism of La on the semiconductor surface. In Figure 1, where the optimized surfaces (101) of ZnTiO_3_ and La/ZnTiO_3_ are shown comparatively, the generation of La-O-Ti bonds on the surface of ZnTiO_3_ can be seen, which supports the experimental results obtained previously. The generation of La-O-Ti bonds not only allows the stabilization of small crystalline particles [79], but it can also generate the change in the structure of bands and states of the surface electrons. To also verify this argument, the DFT calculation was carried out using the optimized La/ZnTiO_3_ model.

### 4.2. Electronic Structure of La/ZnTiO_3_

To better understand the effect of lanthanum doping on the electronic structure of ZnTiO_3_, the electronic band structure diagrams of this semiconductor in both its pure and La-doped forms were plotted. In Figure 2, we can see that the energy levels of ZnTiO_3_ are less dense than those shown for La/ZnTiO_3_, probably because the latter presents hybrid levels or levels of impurities due to the presence of lanthanum. This figure shows that La/ZnTiO_3_ has two impurity energy levels located just above the valence band maximum (VBM) of pure ZnTiO_3_. Likewise, in Figure 2, another evident characteristic of La/ZnTiO_3_ is that the Fermi level (E_F_) crosses the impurity energy levels mentioned above, which, according to the literature, would mean that the E_F_ is not completely full of electrons in the ground state. The hybrid levels or levels of impurities would be forming a surface acceptor in the La/ZnTiO_3_ due to the continuous state that is generated by the smaller distance and overlap between the energy levels of the impurities and the VBM. In this way, the surface acceptor, acting as a trap for photoexcited holes, could allow electrons in the VB to be first excited to isolated impurity energy levels and then excited to the CB. Throughout this photochemical process, the electrons would need less photon energy to be excited, so doping with La would allow the photocatalyst to obtain a better response to visible light. If the energy levels of the impurities are taken into account, the bandgap would be greatly reduced, and the acceptor effect would be more evident as the concentration of lanthanum increases. However, several authors have shown the efficacy of using La ≤ 1–2 wt.% to dope semiconductors and improve their photocatalytic activity in the UV and visible light regions [80]. This is probably due to the fact that the use of high concentrations of lanthanum as a dopant could increase the amount of oxygen vacancies and consequently increase the recombination centers of photoinduced electrons and holes; in addition, the high agglomeration of dopant particles could block the active sites on the catalyst surface and decrease its photoactivity [81,82].

To determine the indirect bandgap value of La/ZnTiO_3_, a Hubbard approximation term was implemented in order to accurately explain the electronic structure of this semiconductor [47]. In a previous study, we reported an indirect bandgap value of 3.16 eV for the ZnTiO_3_ structure. In this study, the indirect bandgap value for the La/ZnTiO_3_ structure was estimated to be 2.92 eV. As can be seen in Figure 2, the bandgap of the semiconductor decreases when La disperses on its surface, and this theoretical result supports the experimental results that we reported previously. The bandgap plays a fundamental role in the photocatalytic activity of semiconductors because it participates in the determination of the *e^-^/h^+^* recombination rate [83]. Therefore, La/ZnTiO_3_ could be more photoactive than ZnTiO_3_ due to the smaller separation between the occupied and unoccupied bands [34]. Table 4 shows the comparison of the bandgap energy values of ZnTiO_3_ and La/ZnTiO_3_ calculated in this study with other experimental energy values reported in the literature.

Regarding the electronic nature of the La/ZnTiO_3_ structure, Figure 3 and Figure 4 show that the hybridization of Zn-O bonds and Ti-O bonds occurs principally in their 3*d* and 2*p* orbitals, while the hybridization of La-O bonds occurs mainly in their 5*d* and 2*p* orbitals. The charge distribution of the Ti-O, La-O, and Zn-O bonds indicates that the empty 3*d* orbital in Ti^4+^ (3*d*^0^) and the nearly empty 5*d* orbital in La^3+^ (5*d*^1^) more easily generate a covalent bond with O atoms than the completely occupied 3*d* orbital in Zn^2+^ (3*d*^10^).

On the other hand, Figure 5 shows the respective contour plots of the charge density difference of ZnTiO_3_ and La/ZnTiO_3_. At the free surface, the electronic structure changes due to the existence of the La atom. ELF analysis allowed a better description of the La-O chemical bond. According to the literature, the areas of charge depletion around the La atom and the areas of charge accumulation around the three O atoms could suggest ionic bonds between the La and O atoms [50]. However, the spherical shells around the nuclei clearly showed that these atoms were separated but still polarized, suggesting a polar covalent bond [76].

### 4.3. MB Adsorption on Surface (101) of ZnTiO_3_ and La/ZnTiO_3_

Numerous experimental studies of methylene blue removal from aqueous solutions have confirmed that this dye can be adsorbed on ZnTiO_3_ surfaces without difficulty, due to the electrostatic attraction between the surface oxygen atoms of the semiconductor and the positive regions of the MB molecule. The results reported in a previous study demonstrated that the MB molecule oriented perpendicular to the ZnTiO_3_ surface was the most favored (*E_ads_* = −282 kJ/mol). In fact, the MB molecule with orientation completely parallel to the surface was slightly bent away from it due to electrostatic repulsion. According to the optimized configurations, the adsorption of MB on ZnTiO_3_ occurred in a bidentate chelating mode through the formation of hydrogen bonds, promoting a highly stable adsorbate–surface interaction [68]. In contrast, the results of this study show that the MB molecule with partially parallel orientation and with the N heteroatom close to La dispersed on the ZnTiO_3_ surface and adsorbed with higher negative energy (*E_ads_* = −201.5 kJ/mol) than in the other orientations. Although theoretically the MB molecular adsorption process on the surface (101) of ZnTiO_3_ is energetically more favorable than on the (101) surface of La/ZnTiO_3_, the experimental evidence that we previously reported suggests that the dispersion of La on the semiconductor surface is an important requirement to continue with the next photocatalytic processes.

In the literature, insufficient computational studies were found about the molecular adsorption of MB on lanthanum dispersed on semiconductors; therefore, in Table 5, the results achieved in this investigation are contrasted with those results reported in the literature for the molecular adsorption of several dyes on ZnTiO_3_, which were calculated by the GGA/PBE method.

#### Proposed Photocatalytic Mechanism

Current investigation has found that diverse counterions can facilitate the dispersion of active sites on the surface of a catalyst. This dispersion increases as the diameter of the counterions increases. La^3+^ is a large diameter counterion and is therefore useful for increasing the SSA of the ZnTiO_3_ catalyst and for dispersing its active sites. La^3+^ has an important electron-withdrawing effect, which helps to generate Lewis acid centers, which provide the material catalytic stability in an aqueous reaction medium [84]. Consequently, the modification of La^3+^ can effectively modify both the SSA as the type of active acid center of a catalyst [85].

In this study, the use of La/ZnTiO_3_ allowed an effective degradation of the methylene blue solution, probably for the following reasons. First, the higher SSA of La/ZnTiO_3_ compared to ZnTiO_3_ would increase the adsorption capacity of the catalyst and provide a more active adsorption center for the target molecule. Second, the dispersion of La^3+^ on the surface of ZnTiO_3_ could enhance the transfer of photoinduced electrons from the bulk to the surface and thus prevent recombination of (*e*^−^/*h*^+^) pairs under the radiation effect [38].

Since it has been suggested that the existence of La ions on the surface of ZnTiO_3_ influences the photocatalytic activity of this semiconductor by altering the recombination rate of the (*e*^−^/*h*^+^) pair, the photocatalytic process in which La/ZnTiO_3_ participates could begin when the electrons (*e*^−^) of the photocatalyst are photoexcited and immediately transferred from the valence band (VB) to the conduction band (CB), leaving a hole (*h*^+^) in the VB (reaction R1) due to the formation of a pair (*e*^−^/*h*^+^). These (*e*^−^/*h*^+^) pairs have the ability to recombine immediately (reaction R2); some of these pairs can even migrate to the semiconductor surface and react separately with species that are adsorbed on the surface, such as H_2_O, OH^−^, O_2_, and other molecules (R), including the MB dye. The holes generated in the VB of the semiconductor are capable of oxidizing adsorbed water molecules or hydroxyl ions to generate strongly reactive hydroxyl radicals (reactions R3 and R4). The lifetime of free (*h*^+^) could be prolonged by allowing more of these holes to diffuse to the ZnTiO_3_ surface to create more reactive radicals that allow oxidizing adsorbed molecules on the surface. On the other hand, a La^3+^ ion with completely empty 4*f* and 5*d* orbitals can confine photoexcited electrons in the CB of ZnTiO_3_ (reaction R5); therefore, the La^3+^ ion becomes a La^2+^ ion by capturing an (*e*^−^), and considering that the generated La^2+^ ion is unstable, the captured electrons could be transferred to oxygenated molecules adsorbed on the surface of ZnTiO_3_, generating hydroxyl radicals (OH˙) and superoxide radical anions (O_2_˙^−^) through a sequence of reactions (reactions R6–R9). It is important to mention that both OH˙ and O_2_˙^−^ radicals can easily oxidize organic molecules. In fact, O_2_˙^-^ has a enough reduction potential to oxidize organic compounds with strong electron-donating groups; nevertheless, the OH˙ radical can remove H atoms or attack unsaturated C-C bonds [38]. Consequently, a model compound containing unsaturated C-C bonds, such as MB, is very likely to be photo-oxidized or attacked by the OH˙ radicals formed (reaction R10). Moreover, direct oxidation of the MB molecule could also occur if it reacts with the holes (reaction R11) [79]. The following reactions suggest the likely route for MB dye photodegradation on the La/ZnTiO_3_ surface [49]:

(R1) $$\left({\mathrm{ZnTiO}}_{3}\right)\overset{\mathrm{hv}}{\to }{\;\mathrm{ZnTiO}}_{3}+e_{\mathrm{CB}}^{-}+h_{\mathrm{VB}}^{+}$$

(R2) $$e_{\mathrm{CB}}^{-}+h_{\mathrm{VB}}^{+}\to \mathrm{heat}$$

(R3) $$H_{2}O_{\mathrm{ads}}+h_{\mathrm{VB}}^{+}\;⇌\;{\left(H^{+}+{\;\mathrm{OH}}^{-}\right)}_{\mathrm{ads}}+h_{\mathrm{VB}}^{+}\to {\mathrm{OH}}_{\mathrm{ads}}^{•}$$

(R4) $${\mathrm{OH}}_{\mathrm{ads}}^{-}+h_{\mathrm{VB}}^{+}\to {\;\mathrm{OH}}_{\mathrm{ads}}^{•}$$

(R5) $${\mathrm{La}}^{3+}+e_{\mathrm{CB}}^{-}\;\to {\;\mathrm{La}}^{2+}$$

(R6) $${\mathrm{La}}^{2+}+{\left(O_{2}\right)}_{\mathrm{ads}}\;\to {\;\mathrm{La}}^{3+}+{\;O}_{2}^{•-}$$

(R7) $$O_{2}^{•-}+{\;H}^{+}\to {\;\mathrm{HO}}_{2}^{•}$$

(R8) $$2{\mathrm{HO}}_{2}^{•}\to {\;H}_{2}O_{2}+{\;O}_{2}$$

(R9) $$H_{2}O_{2}+e_{\mathrm{BC}}^{-}\to {\;\mathrm{OH}}^{•}+{\;\mathrm{OH}}^{-}$$

(R10) $$R+{\;\mathrm{OH}}_{\mathrm{ads}}^{•}\to {\;R}_{\mathrm{ads}}^{\prime •}+{\;H}_{2}O\to \mathrm{degradation}\;\mathrm{products}$$

(R11) $$R_{\mathrm{ads}}+h_{\mathrm{VB}}^{+}\to {\;R}_{\mathrm{ads}}^{•+}\to \mathrm{degradation}\;\mathrm{products}$$

## 5. Conclusions

ZnTiO_3_ is a semiconductor with a wide bandgap; therefore, the biggest problem with using ZnTiO_3_ for MB dye photodegradation lies in the fact that it is only active under UV light radiation. The dispersion of lanthanum (La) on the surface of ZnTiO_3_ could address this problem, since it allows hybrid levels in the VB and the CB to form, decreases the bandgap width, changes surface electron states, reduces the potential energy for MB adsorption, and decreases the energy barriers for MB photodegradation under solar irradiation. The dispersion of La on the surface of ZnTiO_3_ also distorts the surface lattice of the semiconductor, restricting the growth of crystallites to obtain nanometer-sized particles with a higher specific surface area.

La atom could not be intercalated in ZnTiO_3_ body; however, it had the ability to bond with oxygen on the ZnTiO_3_ surface to form a La-O-Ti bond. Furthermore, the electronic structures showed that La-O bonds had both ionic and covalent characteristics; thus, they are polar covalent bonds. Likewise, the lower bandgap energy of La/ZnTiO_3_ (2.92 eV) compared to the bandgap energy of ZnTiO_3_ (3.16 eV) was verified.

Experimental evidence indicates that the La/ZnTiO_3_ photocatalyst exhibits better MB removal capacity from aqueous solutions than the ZnTiO_3_ photocatalyst. In this study, the molecular adsorption process of MB on the surface of La/ZnTiO_3_ with the molecule located in the P_4_ orientation was energetically more favored than in the other orientations. In addition, the negative values of heat cleavage obtained in this study suggest that the incorporation of La on the surface of ZnTiO_3_ is thermodynamically stable. Therefore, based on the literature and evidence from this study, it is suggested that the enhanced removal of MB using La/ZnTiO_3_ would occur through the following mechanism. First, the MB molecule is adsorbed on the surface of La/ZnTiO_3_. This process can happen at room temperature without irradiation. Second, electrons in the VB of La/ZnTiO_3_ can be photoexcited to hybrid levels, mainly composed of La5*d* and O2*p* orbitals, through the band transition [44]. Likewise, the electrons in the CB can also be photoexcited to the catalyst surface through the intraband transition to finally oxidize the MB molecule.

In conclusion, the results of this theoretical study showed that the La/ZnTiO_3_ compound could effectively improve MB dye removal from aqueous solutions, which is consistent with previously obtained experimental results. The results presented in this article confirm the feasibility of using these photocatalysts as excellent dye adsorbents for future environmental applications.

## Figures and Tables

**Table 1:** Significant bond lengths and angles for La/ZnTiO_3_ surface.

Bond Length (Å)		Angle (°)	
Atoms	La/ZnTiO_3_	Atoms	La/ZnTiO_3_
La-O4	2.32	La-O4-Ti17	117.04
La-O6	2.32	La-O6-Ti26	116.25
La-O8	2.38	La-O8-Ti23	133.26

**Table 2:** Adsorption energy and heat segregation for different surfaces.

Position	Bond	∆*E_ads_* (kJ/mol)	∆*G_seg_* (kJ/mol)
ZnTiO_3_	O-H	−64.06	-
La/ZnTiO_3_: P_0_	-	+353.43	-
La/ZnTiO_3_: P_1_	La-S	−82.81	−57.00
La/ZnTiO_3_: P_2_	La-S	−85.44	−57.00
La/ZnTiO_3_: P_3_	La-N	−199.57	−58.19
La/ZnTiO_3_: P_4_	La-N	−201.50	−58.19

**Table 3:** Bader’s charge analysis for the selected atoms at the surfaces.

Absorption System	Atom	Total Electron (-*e*) before Adsorption	Total Electron (-*e*) after Adsorption	Transfer Charge (-*e*)
MB absorbed on ZnTiO_3_	H1	0.89	0.88	+0.02
	H2	0.88	0.86	+0.01
	O1	7.11	7.15	−0.03
	O2	7.11	7.14	−0.03
MB absorbed on La/ZnTiO_3_	N1	7.45	7.77	−0.33
	La	8.85	8.87	+0.02

**Table 4:** Calculated bandgap energy of La/ZnTiO_3_ and other experimental energy values reported in the literature.

Adsorbent	Method	Bandgap (eV)	Reference
ZnTiO_3_/TiO_2_	Experimental	3.07	[78]
La/ZnTiO_3_/TiO_2_	Experimental	3.04	[78]
ZnTiO_3_	Experimental	3.54	[81]
La/ZnTiO_3_ (1%)	Experimental	3.37	[81]
La/ZnTiO_3_ (2%)	Experimental	2.92	[81]
La/ZnTiO_3_ (3%)	Experimental	3.35	[81]
La/ZnTiO_3_ (4%)	Experimental	3.01	[81]
La/ZnTiO_3_ (5%)	Experimental	3.12	[81]
ZnTiO_3_	VASP (GGA/PBE+U)	3.16	[68]
La/ZnTiO_3_	VASP (GGA/PBE+U)	2.98	This study

**Table 5:** Calculated molecular adsorption energy values of MB and other dyes on surfaces (101) of La/ZnTiO_3_ and ZnTiO_3_.

Adsorbent	Dye	Software Used	Adsorption (kJ/mol)	References
ZnTiO_3_	*s*-Cu-TTC	VASP	−296.56	[60]
ZnTiO_3_	TPA-1	CASTEP	−136.39	[63]
ZnTiO_3_	TPA-2	CASTEP	−157.47	[63]
ZnTiO_3_	TPA-3	CASTEP	−561.33	[63]
ZnTiO_3_	TPA-4	CASTEP	−228.19	[63]
ZnTiO_3_ (H)	MB	VASP	−126.76	[68]
ZnTiO_3_ (SP)	MB	VASP	−282.05	[68]
ZnTiO_3_ (P_4_)	MB	VASP	−64.06	This study
La/ZnTiO_3_ (P_1_)	MB	VASP	−82.81	This study
La/ZnTiO_3_ (P_2_)	MB	VASP	−85.44	This study
La/ZnTiO_3_ (P_3_)	MB	VASP	−199.57	This study
La/ZnTiO_3_ (P_4_)	MB	VASP	−201.50	This study

## Data Availability

Data are contained within the article and Supplementary Materials.

## Supplementary Materials

The following supporting information can be downloaded at: https://www.mdpi.com/article/10.3390/nano12183137/s1, Table S1: Relevant interatomic distances for La/ZnTiO_3_ surface; Table S2: Bader’s charge analysis of the optimized ZnTiO_3_ and La/ZnTiO_3_ surfaces.
