# Supplementary material for: The Effect of La3+ on the Methylene Blue Dye Removal Capacity of the La/ZnTiO3 Photocatalyst, a DFT Study
Source: Nanomaterials (Basel). 2022 Sep 10;12(18):3137. doi: 10.3390/nano12183137 (PMC9505429; doi:10.3390/nano12183137)
Supplement: Supplementary file 1 [file nanomaterials-12-03137-s001.zip › nanomaterials-1839998-supplementary.pdf]

Table S1. Relevant interatomic distances for La/$ZnTiO_3$ surface.

Atoms	Bond length (Å)	
	$ZnTiO_3$	La/$ZnTiO_3$
O1 - Ti8	1.68	1.69
O2 - Ti11	1.68	1.69
O3 - Ti14	1.68	1.69
O4 - Ti17	1.69	1.78
O5 - Ti20	1.68	1.69
O6 - Ti26	1.69	1.78
O7 - Ti29	1.68	1.69
O8 - Ti23	1.69	1.78
O9 - Ti32	1.68	1.69
O10 - Ti35	1.68	1.69
O11 - Ti2	1.68	1.68
O12 - Ti5	1.68	1.69

Table S2. Bader`s charge analysis of the optimized $ZnTiO_3$ and La/$ZnTiO_3$ surfaces.

No.	Atom	X	Y	Z	$ZnTiO_3$ Charge (-*e*)	La/$ZnTiO_3$ Charge (-*e*)	Difference Charge (-*e*)
1	O	4.318	3.077	7.030	-1.116	-1.135	-0.019
2	O	13.957	4.695	7.023	-1.115	-1.134	-0.019
3	O	8.600	6.465	7.014	-1.115	-1.147	-0.032
4	O	3.908	9.003	7.023	-1.114	-1.271	-0.157
5	O	13.056	9.744	7.019	-1.112	-1.149	-0.037
6	O	3.038	12.572	6.965	-1.114	-1.289	-0.176
7	O	12.158	14.732	7.003	-1.114	-1.142	-0.028
8	O	7.164	11.404	6.849	-1.113	-1.301	-0.188
9	O	6.850	16.305	6.968	-1.113	-1.154	-0.041
10	O	1.690	18.028	6.995	-1.115	-1.137	-0.022
11	O	11.262	19.817	7.003	-1.113	-1.128	-0.015
12	O	9.566	1.234	7.003	-1.114	-1.131	-0.017
13	O	4.719	5.133	4.541	-1.327	-1.339	-0.012
14	O	0.360	1.752	4.539	-1.327	-1.330	-0.003
15	O	9.969	3.442	4.540	-1.328	-1.332	-0.004
16	O	3.827	10.203	4.541	-1.326	-1.374	-0.049
17	O	-0.532	6.823	4.539	-1.327	-1.337	-0.010
18	O	9.077	8.513	4.540	-1.327	-1.335	-0.008
19	O	2.935	15.273	4.541	-1.327	-1.345	-0.018
20	O	-1.423	11.893	4.539	-1.327	-1.336	-0.010
21	O	8.185	13.583	4.540	-1.325	-1.331	-0.006
22	O	5.611	0.063	4.541	-1.328	-1.333	-0.004
23	O	-2.315	16.963	4.539	-1.326	-1.330	-0.004
24	O	7.294	18.653	4.540	-1.328	-1.332	-0.004
25	O	10.765	6.091	4.665	-1.321	-1.327	-0.006
26	O	6.407	2.710	4.665	-1.321	-1.326	-0.005
27	O	1.157	4.400	4.665	-1.321	-1.328	-0.007
28	O	11.656	1.021	4.665	-1.320	-1.324	-0.004
29	O	5.515	7.780	4.665	-1.321	-1.326	-0.005

30	O	0.265	9.470	4.665	-1.320	-1.337	-0.018
31	O	4.623	12.850	4.665	-1.320	-1.375	-0.054
32	O	-0.627	14.540	4.665	-1.322	-1.335	-0.013
33	O	9.873	11.161	4.665	-1.321	-1.346	-0.025
34	O	3.731	17.921	4.665	-1.321	-1.325	-0.005
35	O	-1.519	19.610	4.665	-1.321	-1.324	-0.004
36	O	8.981	16.231	4.665	-1.320	-1.328	-0.009
37	O	12.926	4.321	4.127	-1.335	-1.337	-0.002
38	O	8.568	0.940	4.128	-1.334	-1.335	-0.001
39	O	3.318	2.629	4.127	-1.334	-1.337	-0.002
40	O	12.034	9.392	4.127	-1.335	-1.338	-0.003
41	O	7.676	6.010	4.128	-1.334	-1.337	-0.003
42	O	2.426	7.699	4.127	-1.334	-1.355	-0.021
43	O	11.143	14.462	4.127	-1.335	-1.336	-0.001
44	O	6.784	11.081	4.128	-1.335	-1.341	-0.007
45	O	1.535	12.769	4.127	-1.333	-1.341	-0.007
46	O	10.251	19.532	4.127	-1.335	-1.336	-0.001
47	O	5.893	16.151	4.128	-1.335	-1.334	0.000
48	O	0.643	17.839	4.127	-1.333	-1.335	-0.002
49	O	13.106	1.145	2.598	-1.307	-1.308	-0.001
50	O	7.856	2.833	2.598	-1.307	-1.308	-0.001
51	O	2.607	4.522	2.597	-1.307	-1.310	-0.003
52	O	12.215	6.215	2.598	-1.307	-1.308	-0.002
53	O	6.965	7.903	2.598	-1.307	-1.312	-0.005
54	O	1.715	9.592	2.597	-1.307	-1.312	-0.005
55	O	6.073	12.973	2.598	-1.307	-1.313	-0.006
56	O	11.323	11.285	2.598	-1.307	-1.309	-0.002
57	O	10.431	16.355	2.598	-1.306	-1.308	-0.002
58	O	5.181	18.043	2.598	-1.307	-1.309	-0.002
59	O	-0.069	19.732	2.597	-1.307	-1.309	-0.002
60	O	0.823	14.662	2.597	-1.307	-1.310	-0.003
61	O	11.837	3.633	1.708	-1.367	-1.368	0.000
62	O	11.564	1.221	0.245	-1.179	-1.181	-0.002
63	O	5.128	1.515	2.237	-1.330	-1.330	0.000
64	O	4.050	4.200	0.424	-1.182	-1.184	-0.002
65	O	7.479	0.251	1.709	-1.367	-1.367	0.000
66	O	6.317	2.905	0.248	-1.179	-1.181	-0.002
67	O	-0.120	3.209	2.237	-1.331	-1.329	0.002
68	O	14.547	0.823	0.423	-1.181	-1.182	-0.001
69	O	2.228	1.942	1.708	-1.367	-1.368	0.000
70	O	1.065	4.601	0.245	-1.179	-1.180	-0.002
71	O	9.488	4.897	2.237	-1.331	-1.330	0.001
72	O	9.297	2.512	0.423	-1.181	-1.183	-0.002
73	O	10.946	8.703	1.708	-1.367	-1.369	-0.001
74	O	10.672	6.291	0.245	-1.179	-1.181	-0.002
75	O	4.236	6.585	2.237	-1.330	-1.331	-0.001
76	O	3.158	9.270	0.424	-1.182	-1.187	-0.005
77	O	6.587	5.321	1.709	-1.367	-1.368	-0.001
78	O	5.425	7.976	0.248	-1.179	-1.180	-0.001

79	O	-1.012	8.279	2.237	-1.331	-1.329	0.002
80	O	13.655	5.893	0.423	-1.181	-1.183	-0.001
81	O	1.336	7.012	1.708	-1.367	-1.369	-0.002
82	O	0.173	9.671	0.245	-1.179	-1.182	-0.003
83	O	8.596	9.967	2.237	-1.330	-1.332	-0.001
84	O	8.405	7.583	0.423	-1.181	-1.182	-0.001
85	O	10.054	13.774	1.708	-1.367	-1.369	-0.001
86	O	9.781	11.361	0.245	-1.179	-1.181	-0.002
87	O	3.345	11.655	2.237	-1.330	-1.330	0.001
88	O	2.266	14.341	0.424	-1.182	-1.184	-0.003
89	O	5.695	10.391	1.709	-1.367	-1.374	-0.006
90	O	4.533	13.046	0.248	-1.180	-1.181	-0.001
91	O	-1.903	13.349	2.237	-1.331	-1.328	0.003
92	O	12.763	10.963	0.423	-1.181	-1.182	-0.001
93	O	0.444	12.082	1.708	-1.367	-1.370	-0.002
94	O	-0.719	14.741	0.245	-1.179	-1.181	-0.002
95	O	7.704	15.037	2.237	-1.331	-1.332	-0.002
96	O	7.513	12.653	0.423	-1.181	-1.184	-0.003
97	O	9.162	18.844	1.708	-1.367	-1.368	-0.001
98	O	8.889	16.431	0.245	-1.179	-1.181	-0.002
99	O	2.453	16.726	2.237	-1.330	-1.330	0.000
100	O	1.374	19.411	0.424	-1.182	-1.183	-0.002
101	O	4.803	15.461	1.709	-1.367	-1.370	-0.003
102	O	3.641	18.116	0.248	-1.179	-1.181	-0.002
103	O	-2.795	18.419	2.237	-1.331	-1.330	0.001
104	O	11.871	16.033	0.423	-1.181	-1.183	-0.001
105	O	-0.448	17.152	1.708	-1.367	-1.368	-0.001
106	O	-1.611	19.811	0.245	-1.179	-1.180	-0.002
107	O	6.812	20.108	2.237	-1.331	-1.329	0.001
108	O	6.621	17.723	0.423	-1.181	-1.182	-0.001
109	Zn	9.311	1.471	2.148	1.325	1.323	-0.002
110	Zn	0.790	2.602	0.429	1.214	1.208	-0.006
111	Zn	3.660	0.589	4.267	1.299	1.293	-0.005
112	Zn	4.063	3.161	2.150	1.325	1.322	-0.003
113	Zn	10.398	4.292	0.429	1.214	1.208	-0.005
114	Zn	13.268	2.281	4.267	1.300	1.296	-0.004
115	Zn	13.670	4.853	2.149	1.325	1.323	-0.002
116	Zn	6.041	0.910	0.429	1.215	1.208	-0.006
117	Zn	8.019	3.970	4.267	1.300	1.295	-0.005
118	Zn	8.419	6.541	2.148	1.325	1.323	-0.003
119	Zn	-0.102	7.673	0.429	1.214	1.208	-0.006
120	Zn	2.769	5.659	4.267	1.300	1.273	-0.027
121	Zn	3.171	8.231	2.150	1.325	1.315	-0.010
122	Zn	9.506	9.363	0.429	1.214	1.207	-0.006
123	Zn	12.376	7.351	4.267	1.301	1.296	-0.005
124	Zn	12.778	9.923	2.149	1.325	1.321	-0.004
125	Zn	5.149	5.980	0.429	1.215	1.207	-0.008
126	Zn	7.127	9.040	4.267	1.299	1.279	-0.020
127	Zn	7.527	11.611	2.148	1.326	1.320	-0.006

128	Zn	-0.994	12.743	0.429	1.214	1.208	-0.006
129	Zn	1.877	10.729	4.267	1.302	1.287	-0.015
130	Zn	2.279	13.301	2.150	1.325	1.317	-0.008
131	Zn	8.615	14.432	0.429	1.214	1.206	-0.007
132	Zn	11.484	12.421	4.267	1.301	1.293	-0.008
133	Zn	11.886	14.993	2.149	1.325	1.321	-0.004
134	Zn	4.257	11.050	0.429	1.215	1.205	-0.010
135	Zn	6.235	14.110	4.267	1.301	1.282	-0.019
136	Zn	6.635	16.681	2.148	1.325	1.322	-0.003
137	Zn	-1.886	17.813	0.429	1.214	1.207	-0.007
138	Zn	0.985	15.800	4.267	1.301	1.293	-0.008
139	Zn	1.388	18.372	2.150	1.325	1.322	-0.002
140	Zn	7.723	19.503	0.429	1.214	1.208	-0.006
141	Zn	10.593	17.491	4.267	1.301	1.299	-0.003
142	Zn	10.994	20.063	2.149	1.325	1.323	-0.002
143	Zn	3.365	16.120	0.429	1.215	1.206	-0.009
144	Zn	5.343	19.181	4.267	1.300	1.298	-0.002
145	Ti	12.871	2.166	1.038	2.515	2.509	-0.006
146	Ti	-3.244	20.151	5.389	2.512	2.512	0.000
147	Ti	6.624	1.259	3.365	2.593	2.587	-0.006
148	Ti	7.622	3.854	1.038	2.515	2.508	-0.007
149	Ti	9.930	1.560	5.390	2.511	2.513	0.002
150	Ti	1.375	2.950	3.364	2.594	2.588	-0.006
151	Ti	3.263	0.475	1.039	2.515	2.507	-0.008
152	Ti	4.682	3.248	5.390	2.514	2.510	-0.004
153	Ti	10.983	4.640	3.365	2.594	2.587	-0.006
154	Ti	11.979	7.236	1.038	2.515	2.507	-0.007
155	Ti	-0.569	4.941	5.389	2.514	2.511	-0.003
156	Ti	5.732	6.329	3.365	2.593	2.558	-0.035
157	Ti	6.731	8.924	1.038	2.515	2.502	-0.014
158	Ti	9.039	6.630	5.390	2.513	2.507	-0.006
159	Ti	0.483	8.020	3.364	2.594	2.570	-0.025
160	Ti	2.371	5.546	1.039	2.515	2.507	-0.008
161	Ti	3.790	8.318	5.390	2.513	2.402	-0.111
162	Ti	10.091	9.711	3.365	2.593	2.567	-0.026
163	Ti	11.087	12.306	1.038	2.515	2.505	-0.010
164	Ti	-1.461	10.011	5.389	2.511	2.512	0.001
165	Ti	4.841	11.399	3.365	2.593	2.500	-0.093
166	Ti	5.839	13.994	1.038	2.515	2.497	-0.018
167	Ti	8.147	11.700	5.390	2.511	2.428	-0.084
168	Ti	-0.408	13.091	3.364	2.594	2.569	-0.025
169	Ti	1.479	10.616	1.039	2.515	2.495	-0.020
170	Ti	2.898	13.388	5.390	2.513	2.407	-0.107
171	Ti	9.199	14.781	3.365	2.593	2.563	-0.030
172	Ti	10.196	17.376	1.038	2.515	2.508	-0.006
173	Ti	-2.352	15.081	5.389	2.513	2.513	0.000
174	Ti	3.949	16.469	3.365	2.593	2.573	-0.019
175	Ti	4.947	19.065	1.038	2.515	2.508	-0.008
176	Ti	7.255	16.770	5.390	2.511	2.516	0.005

177	Ti	-1.300	18.161	3.364	2.594	2.587	-0.007
178	Ti	0.587	15.686	1.039	2.515	2.504	-0.011
179	Ti	2.006	18.458	5.390	2.513	2.512	-0.001
180	Ti	8.307	19.851	3.365	2.594	2.587	-0.007
181	La	4.833	11.123	7.213	-	2.150	2.150
